# In the presence of a patent foramen ovale paroxysmal embolism risk increases with non-vortical right atrial blood flow

**DOI:** 10.1186/1532-429X-18-S1-P227

**Published:** 2016-01-27

**Authors:** Victoria Stoll, Aaron T Hess, Oliver Rider, Hayley Harvey, Alex Pitcher, Margaret Loudon, Malenka M Bissell, Stefan Neubauer, Oliver Ormerod, Saul G Myerson

**Affiliations:** 1 grid.4991.50000000419368948OCMR, University of Oxford, Oxford, United Kingdom; 2 grid.8348.70000000123067492John Radcliffe Hospital, Oxford, United Kingdom

## Background

Despite the fact that stroke is a leading cause of disability, in up to 40% of cases no cause is found on routine clinical investigation. Although a patent foramen ovale (PFO) is an attractive mechanism to explain these cryptogenic strokes, using current imaging techniques, distinguishing between a causative rather than an incidental PFO remains elusive. We hypothesised that, in the presence of a PFO, non-vortical right atrial (RA) flow patterns would be linked to embolism risk by making it more likely to shunt blood, and as such thrombus through the PFO. In order to investigate this we assessed RA flow patterns and interatrial shunt size in patients with a PFO and investigated whether these metrics predicted the incidence of paradoxical embolism.

## Methods

3 groups of participants were recruited to the study; 1) patients with presumed paradoxical embolism via their PFO (n = 20) 2) subjects with a PFO but no embolism (n = 12) and 3) controls without a PFO (n = 28). All underwent RA 4D flow assessment, and bubble transthoracic echocardiography to determine interatrial shunt size.

## Results

**Flow Patterns**

**Figure 1:**
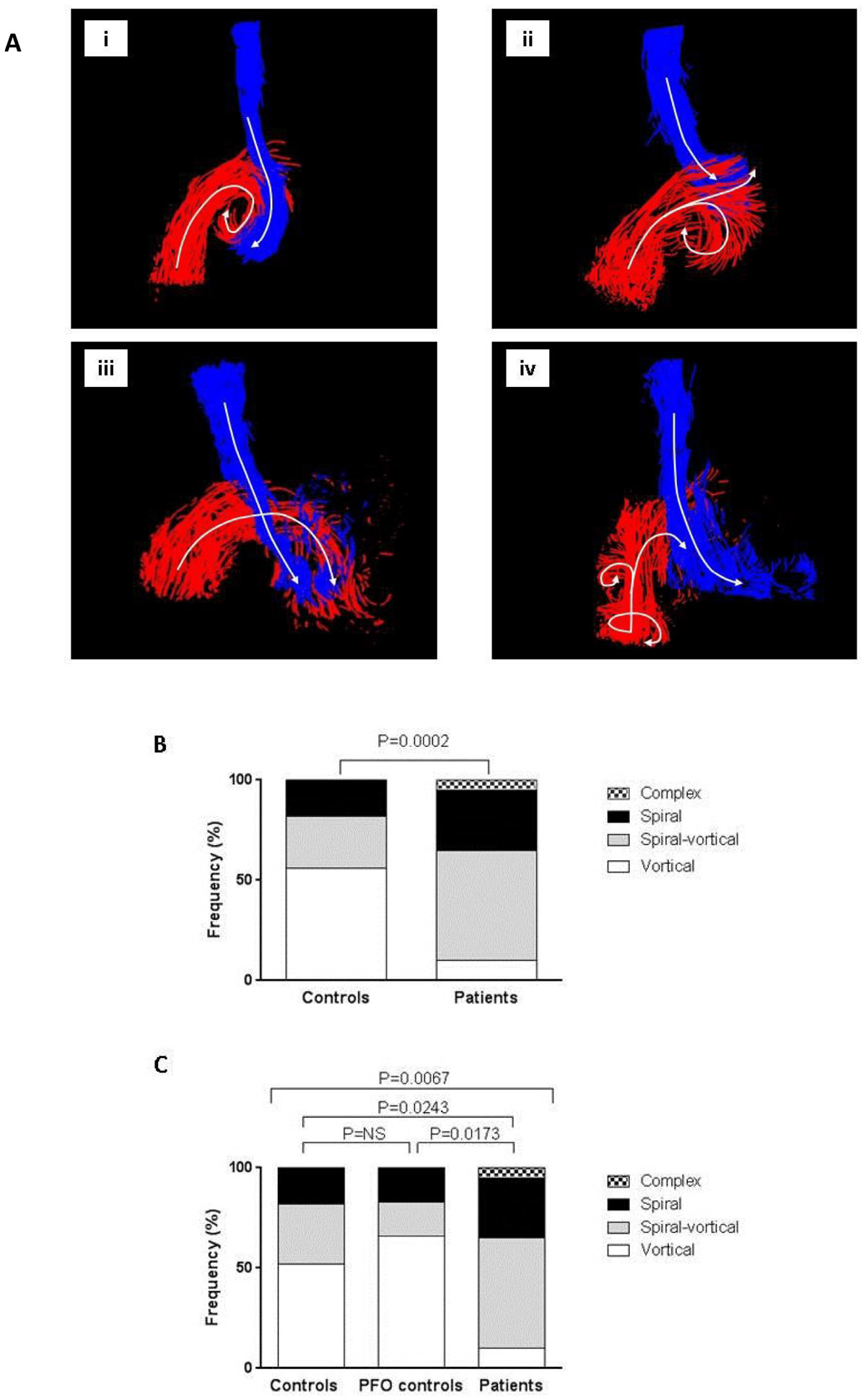
**A.i) Vortical flow: the IVC and SVC turn in a clockwise vortex**. All other flow patterns were classified as non-vortical (abnormal) flow, within this category 3 distinct patterns were identified: ii) **Spiral-vortical flow**: the IVC forms a vortex whilst the SVC passes laterally and is then enveloped in a spiral fashion by the IVC, iii) **Spiral flow**: where the IVC and SVC combine in a spiral, iv) **Complex flow**: involving multiple vortices arising from the IVC and SVC flow. **Figure 1B.** Right atrial flow patterns in controls compared to patients with a PFO. **Figure 1C.** Right atrial flow patterns in controls without a PFO, compared to controls with a PFO but no embolic event and patients with a PFO and presumed embolic event.

RA flow patterns were significantly different in participants with an embolic event compared to those without, with more non-vortical flow seen in embolic patients (P <0.001, Figure 1A). In addition, whilst flow patterns were similar between non PFO controls and subjects with PFO but no embolic event, they were both different from that seen in PFO patients with embolic events, again with higher incidence of non-vortical RA flow (P=0.0067, Figure 1B).

**Figure 2:**
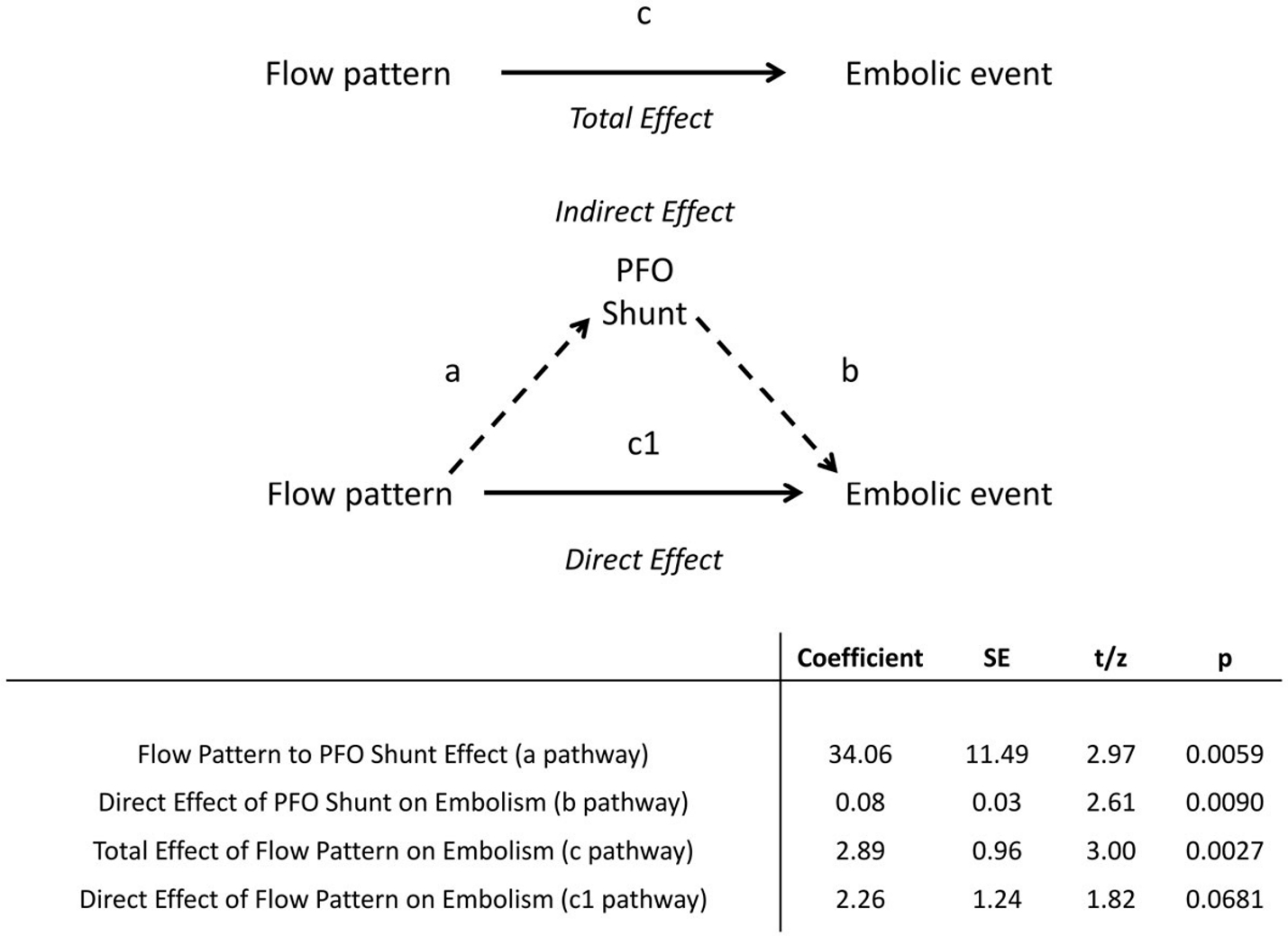
**The contribution of PFO shunt severity to non-vortical flow pattern's influence on embolic event occurrence**.

**Risk of embolism**

When considering all the subjects with a PFO (n = 32), the presence of a non-vortical flow pattern was 11.5 times more common in those who have had an embolic event (P= 0.002, Fisher's exact test). To explore whether this effect was mediated by changing the degree of shunting, moderated multiple regression was performed. This showed that flow patterns were related to shunt grade (a pathway, β 34.0, p <0.01), and that shunt grade was related to embolism incidence (b pathway, β 0.08, p <0.01). As the a and b pathways were significant, mediation analysis was tested using 2000 bootstrap resamples to generate a 95% confidence interval (bias corrected) of the indirect effect. This showed that the effect of an abnormal flow pattern upon an embolic event is indeed mediated by increasing the shunt across the PFO (CI 0.45-18.42, Figure 2). As the direct effect of flow patterns on embolic risk becomes insignificant (c1 pathway, p =0.06) this suggests full mediation.

## Conclusions

Patients with a PFO and a non-vortical RA flow pattern were 11.5 times more likely to have had an embolic event. This increased embolic risk seems to be mediated via increasing the shunt size across the PFO. As a result, not only will identification of the presence or absence of RA vortical flow in individuals presenting with a cryptogenic stroke help distinguish a causative PFO, but it may also identify patients with a PFO who are at elevated risk of future embolism.

